# What, if anything at all, do African societies themselves owe to their own citizens in terms of health?

**DOI:** 10.1186/s12939-022-01822-1

**Published:** 2023-01-06

**Authors:** Thierry Ngosso

**Affiliations:** 1 University of St. Gallen, St. Gallen, CH Switzerland; 2 Catholic University of Central Africa, Yaoundé, CMR Cameroon; 3 The University of Maroua, Maroua, CMR Cameroon

**Keywords:** Global justice, Human right to health, Sub-Saharan Africa, Potential ability

## Abstract

The debate about global justice and health has focused so far on what developed countries owe to developing countries to advance global public health. Less attention has however been paid to the health obligations of developing countries, especially in Sub-Saharan Africa, towards their own people and how this may affect considerations about justice and health in a globalized world. This paper challenges the implicit presumption in global justice theories that African societies, because they are poor, have weaker health obligations toward their own peoples. It makes two main claims. First, despite their economic shortcomings, African governments should have the primary responsibility to protect the domestic side of the human right to health of their own citizens and dumping their own health obligations on rich countries is a disservice to the overall goal of global justice in health. Second, the health obligations of African societies towards their own people should be assessed and grounded also on their *potential* abilities, and not exclusively on their *current* abilities. Global justice in health cannot be reduced to what rich countries should do. It must include also what developing countries from Sub-Saharan Africa should do beyond accepting or managing any health assistance.

## Introduction

In March 12, 2016, the Cameroon public and observers abroad were shocked by the circumstances of Monique’s death in Douala [1], 4 years after another similar incident – Vanessa’s baby vanished from the Ngousso Hospital in Yaoundé [2]. Monique was pregnant with twins. On the day of the delivery, she went to La Quintinie Hospital, the main public hospital of Douala, to give birth and was denied medical assistance because she could not afford the health care costs associated with it. Monique *literally* died on the sidewalk, right at the gate of La Quintinie Hospital. Although her twins were still alive, her sister who opened her womb with an ordinary blade in the end could not save them. All this happened under the the watch of health care workers and La Quintinie Hospital officials as well as some anonymous pedestrians. Monique and her twins died because they were denied medical assistance. They were denied medical assistance because they were too poor to pay for it. Most of us would agree that it was not only an unfortunate, but also an outrageous situation.

One may find it shocking that even the health care personnel did not apply the professional duty to care while Monique’s sister was trying to save her twins. But it would be simplistic considering that the wrongdoers are those who find themselves at the last link in a much longer chain of individual and institutional interactions within the Cameroonian health care system. A more thoughtful approach will recognize that this type of tragedy only reveals the deeper failures of the Cameroonian health system, and more broadly of most African health care systems. It also highlights the disparate inequalities between most African countries and more developed countries.

On the one hand, it is well documented today how broken many African health systems are. The African continent combines the highest mortality rate with the least amount spent per capita on health expenses [3]. For instance, few African countries have lived up to their commitment to spent 15% of their GDP on health expenses following the Abuja declaration [4]. It is no surprise in such a context then that many African citizens rate their health or health care as the lowest in the world [5]. On the other hand, there is no dispute that there are persistent huge health inequalities between African countries and more developed countries, whether it concerns maternal mortality [6], life expectancy [7], access to healthcare personnel [8] or, among other things, health budgets [9].

Faced with these two facts, the crucial question that arises is that of knowing who is responsible for ensuring a form of global equity in health and making access to health care effective for all. That question is even more crucial if one assumes that health is not an aspirational but an authentic human right [10] that must be guaranteed to all and therefore also to all African citizens. Considering health as a human right provides the normative foundation that can help in capturing the moral intuition stating that human beings should not be discriminated against based on geographical origin as far as equal access to basic healthcare is concerned. It also extends the responsibility for ensuring that such equal access to healthcare falls on the entire human community or the so called ‘international community’ as a whole. If this is a step in the good direction, it is not the whole story. Assuming that health is a human right and that African citizens are entitled to that fundamental right, on who does the primary responsibility to make it effective for the citizens of African states rest?

There are at least two ways to approach the issue of identifying the moral agents who should make the human right to health effective for African citizens. To clarify these two approaches, consider Peter Singer’s thought experiment about the drowning child [11]. Faced with this scenario, political philosophers have been mostly concerned with the questions to know what we should do for the drowning child or who should bear the responsibility to save the drowning child? When the question is framed that way, what matters most is the right the drowning child has to be rescued no matter who the rescuer should be. However, there is a second set of questions that are equally important but have been mostly ‘ignored’ by mainstream political theory, including the question concerning why so many children are drowning in the first place in many African societies. When the question is primarily framed that way, it points to the possibility of identifying who from the perspective of African societies bears the responsibility from preventing such a situation.

The first approach has predominantly presented rich countries as the primary duty-bearers of global health equity. Gillian Brock puts it well when she writes:

“… how should we allocate responsibilities for moving toward instantiating improvements in practices, policies and institutions that make progress toward our goals of improving global health? Who is responsible for making changes? Who, in short [ …] is responsible for showing moral leadership in initiating change? Here I argue that a large share of responsibility falls on the global advantaged to make necessary reform [ …]. Typically that means that governments of affluent developed countries have some key responsibilities” [12].

In this configuration, many African societies will have a very small share of responsibility, i.e. few or no strong health obligations at all.

Brock’s framing of global (health) justice fits into a larger conversation on global justice where most accounts, whether they are nationalist, cosmopolitan or Rawlsian, tend to focus on the obligations of rich/developed countries toward poor/developing countries. If global justice is mostly framed as what rich countries owe to poor countries, it is because rich countries are the ‘only’ entities with capacity to assist [13]. Moreover, that duty to assist does not rest exclusively on charitable/aid motives. It is more importantly a strong moral obligation. It arises from the fact that rich countries have set up a coercive international order, both economically and politically, that harm poor countries and prevents them from building the capacities necessary to secure by themselves the fundamental rights of their own people, including the human right to health [14].

There can be good reasons to uphold such approach. When it comes to global health inequalities, it is believed that access to basic health care needs such as surviving childbirth or having access to critical medicines ‘should not depend on where a person is born’ [15]. Moreover, it should not matter where the help to save the drowning child comes from. And that is one of the appealing aspects of thinking of health [16] as a human right because it also extends the spectrum of the duty-bearers beyond one’s own government. I assume in this article that health is a basic and fundamental human right recognized in key legal documents [17] and that there is a wide variety of philosophical arguments about the normative grounding for, and content of, human rights that converge on support for some version of a right to health/care [18] with very few exceptions [19].

The first approach certainly has its moral relevance, but it does not capture the entire complexity of the global health justice debate. Looking at the second approach helps addressing global health inequalities from another standpoint that includes the specific contribution or responsibility of African societies in advancing global health justice. Even if both approaches can be at some point intertwined, the focus of this paper is as much as possible on the share of health responsibilities that fall on African societies themselves when it comes to achieving global health equity. My main position is that that share is larger than what is often recognized in current literature.

This paper makes two claims. First, African societies should stop dumping their primary responsibility to protect the human right to health of their own people on rich countries. Doing so is not only unfair, but it also prevents us from significantly reducing health inequalities and effectively achieving global health justice. I refer to this here as the ‘Duty-Dumping Argument’ (section I). Second, the obligations of African societies should be assessed and grounded on their *potential* abilities too, and not exclusively on their *current* abilities. I refer to this here as the ‘Potential Ability Argument’ (section II).

### The ‘duty-dumping argument’

The ‘Duty-Dumping Argument’ (DDA) claims that it is morally objectionable and practically inefficient to achieve global health justice for African countries by dumping their primary health obligations on rich countries. We cannot reduce significantly global health inequalities and achieve global health justice if health obligations are wrongly and inadequately distributed between rich and poor countries, and in this case if African societies are not effectively those with the primary responsibility to provide for the *basic* healthcare needs of their own people.

The DDA as I distill here is in two parts. On the one hand, I show why duty-dumping is both wrong and inefficient. On the other hand, I suggest a way to avoid duty-dumping by African societies on rich countries. That second part has three steps. The first step attempts to clearly separate as much as possible domestic from global health issues. The second step presents the need for a division of moral labor that is built upon two principles: the functional principle and the capacitarian principle. The third step suggests that an adequate allocation of moral responsibility should give priority to the functional principle over the capacitarian principle on domestic health issues, and priority to the capacitarian principle over the functional principle on global health issues. This will ensure clarity on what African societies should be responsible for and prevent any duty-dumping on their part on other entities.

I borrow the ‘duty-dumping’ term from Buchanan and Decamp [20]. The term voices the concern that an inadequate allocation of moral responsibility will prevent us from achieving global health equity. An allocation of moral responsibility is inadequate and therefore morally objectionable if it provides no solid moral justification to assign a specific health obligation or if it assigns indeterminate obligations to the wrong entity or if the obligation it assigns is too onerous for a moral agent to carry it out [21]. This can take place only in a context where there is a need for a wide range of agents with distinctive functions and specific capacities to achieve specific social or global goals. When health obligations are wrongly assigned, are too onerous to execute or lack sound moral justification, this can do more harm than good to the achievement of noble goals such as global health equity.

According to Buchanan and Decamp, a typical example of an inadequate division of moral labor in the domain of global health is “the claim that pharmaceutical companies that produce antiretroviral drugs have the duty to supply these drugs to all those who could benefit from them at price they can afford” [22]. Such a strong moral obligation lacks solid moral justification: “There is no more reason to believe that drug companies are responsible for providing drugs to all who need them or that for-profit hospital are to provide care to all that need it, than there is to believe that grocers have an obligation to ensure that no one goes without sufficient food” [23].

Unlike Buchanan and Decamp who compare the distribution of duties between states and firms, I compare the distribution of duties between developed/rich and developing/poor countries which many African are. Nevertheless, I suggest that here too there is a kind of duty-dumping scenario taking place between rich and poor countries that prevent us from achieving effective global health justice. The ‘can implies ought’ principle is applied both in a direct and a reverse way. For instance, it could be assumed based on the ‘can implies ought principle’ that because poor countries are incapable of meeting their health obligations, they should have little if not no moral responsibility to do so. The same may apply again that because rich countries have the capacity to address global health issues, they have the moral obligation to do so. The focus is essentially on the (in) capacity of those actors, and not also on their respective roles in different settings. The consequence is that there is no significant progress in terms of equal access to health care, and health inequalities between poor and rich countries continue to be of great concern. If we should be careful to not dump on business corporations’ health duties that should adequately fall onto States simply because they are more and more wealthy and influent, we should also be careful to not dump on rich countries health duties that should be poor countries’ primary responsibility simply because they are very rich. It will be unfair vis-à-vis rich countries to defer their part to contribute to global health justice [24] if African and developing countries are not taking their own share of responsibility for global health seriously by meeting the basic health obligations towards their own people that are within their reach.

If duty-dumping is therefore wrong because it is unfair and does not effectively address global health inequalities, how can we avoid it? I consider three steps to attempt avoiding any duty-dumping by African societies to rich countries that will also help better define the scope of their specific health obligations.

The first step is the necessary distinction between global and domestic health issues. I consider Monique-like cases as domestic health issues that are within the reach of each individual state. There are at least two criteria that help identify domestic health issues. The first is a) their local nature. The second is that b) those health issues can be properly addressed without any significant external intervention and at a relatively minimal cost by local authorities. Global health issues are however a) transboundary health challenges like pandemics, effects of climate change, pollution or wars on health and b) there are health issues that cannot be properly and effectively addressed without the cooperation of more than one state. Such challenges require some institutional innovation that can only be achieved through international cooperation.

If it is central to separate global and domestic health issues, marking a clear delineation between these two sets of issues can however become an enormous normative challenge given the overlapping nature of the domestic and the global today. On the one hand, some local health issues can have the appearance of being domestic but depend on external intervention to be carried out properly. Maternal mortality is certainly a health issue that can be significantly prevented if the right protocols are set up (frequency of pre-natal consultations, proper screening of the evolution of the fetus, etc.). But one could also argue that setting up the right frequency of pre-natal consultation or even administering proper screening of the fetus at the right time requires both the availability of a substantial number of trained medical personnel which is often lacking locally or enough medical equipment which are generally out of the reach of many African countries’ budgets. Malaria is also a local health issue that can be dealt with if there is a minimum of spaying in the neighborhood and the distribution of mosquito nets to vulnerable people and populations. But here also the availability of mosquito nets depends on the limited health budgets of local African governments and urban policies that get insufficient funding. A plausible case can therefore be made that the very reason some African governments cannot guarantee childbirth survival or significantly reduced maternal mortality for instance is because they are politically or economically weakened by some actions or inactions from rich countries [25]. Moreover, even local health issues like insufficient health budget by African governments can be of global concern because they add to statistics on global health inequalities that would tend to suggest that the solution to certain inequalities is first global when it is primarily domestic. Some African governments will for example put more money into buying military material than on building hospitals, hiring health care workers, or making drug affordable for their people. But this comparison will not show up clearly in global health budgets statistics.

On the other hand, some health issues can have the appearance of being global when they are in fact local or intertwined with local situations. Consider again the case of maternal mortality. It is a key part of global health discourses because statistics show huge inequalities between mostly rich and mostly poor countries. One may think therefore that it is because some countries are poor that they cannot properly address the issues to prevent huge maternal mortality in their respective countries. But the fact that poor countries like Cuba [26], Botswana, Rwanda or Tanzania [27] have succeeded at some point to develop a local health care system that properly addresses those issues locally suggests that maternal mortality is more a local than a global health issue.

Consider also for instance the Covid-19 pandemic. It is/was of course a global health crisis [28] because of the way it impacted the entire globe and requested international cooperation to address it. But it is/was also a highly local issue in terms of how local data informed local responses. Many countries closed their borders or significantly reduced the entry of citizens and non-citizens, the local rate of infection determined the configuration of how to tighten or loosen the lockdown policies. It is more and more evident today, especially in the case of most African countries, that global policies in this domain were not backed up by local evidence but by conjectures and false projections about Africa [29].

The least one can say is that it is difficult, but not impossible to clearly separate, as much as we can, domestic from global health issues. While acknowledging some practical nuances and for the sake of a fair distribution of health responsibilities between developing and developed countries and the purpose of ascribing determinate and justifiable obligations, we should attempt or at least postulate the need for such a delineation. Moreover, we should remain flexible to adjust which issue falls into which camp depending on relevant circumstances. For example, for few months, the covid-19 was a local ‘Chinese’ health issue. It later became an international and global issue when the virus spread throughout the planet. It has become more of a local issue again for some countries. Likewise, Ebola has been predominantly a local health issue in few African countries, but of global importance to prevent it from spreading throughout the world.

Once we acknowledge the need to clearly separate as much as possible domestic from global health issues, it is important to recognize the necessity of a division of moral labor that rests upon two principles: the functional principle and the capacitarian principle. The functional principle considers the allocation of moral responsibility to be based upon roles and functions of moral agents while the capacitarian principle allocates responsibility according to the capacity or power of moral agents.

Each of those two principles rest on solid moral ground. Let us consider the functional principle first. Each society, domestic or global, is made up of multiple social institutions or social agents with distinctives roles and capacities. For any society to function at a minimum desirable level and to achieve general goals like social justice for instance, we need several institutions like schools, business corporations, NGOs, States, hospitals, Universities, fire-fighter departments, etc. We know that each of those institutions cannot achieve alone the overall goal of social justice. They need each other to collaborate to do so. We need hospitals to take care of the health of the public, we need schools and universities to cover the intellectual formation of people, we need business corporations to create wealth, or NGOs to pursuit private interests that serve the public good in several domains, etc. The division of *social* labor in that sense is necessary at least because no institution alone can take up all these roles. If that social division of moral labor is necessary, the only way to preserve it at the social level is therefore also to match our moral expectations regarding each of those distinctive institutions with their social roles, i.e., the division of *moral* labor. A suitable division of moral labor cannot ignore the functions and roles of social institutions.

Consider now the capacitarian principle. Besides the functions or roles social institutions play in society, they develop or have (irrespective of their roles) also specific capacities that call for specific moral responsibility. As such we think that schools have the moral obligation to contribute substantially to the intellectual formation of children and students because they are equipped to do so properly (they have teachers, rooms for teacher/students’ exchanges, books, etc.). We do not expect schools primarily to create wealth like business corporations and advance health or health care like hospitals simply because they lack the capacity to do so. Moreover, social institutions also have the capacity or power that goes beyond the perimeter of what their functions give them the competence to do. An interesting example here is the multinational corporation. Their impact in shaping the current globalization and the norms that go with it give it an out-sized influence in many domains that are not strictly economic [30]. As such our moral expectations regarding such institutions cannot be restricted to their roles as private entities, but potentially as political actors too.

If it appears that both the role/function and the capacity/power are two legitimate sources of moral responsibility, any thoughtful division of moral labor that wants to avoid duty-dumping should not only acknowledge both, but more importantly examine how to best combine them. In this respect, we can have three scenarios. The first will rely predominantly on a *functional* division of moral labor where our moral expectations are based mostly on the roles each social institution should play – this can be the kind of division of labor Buchanan and Decamp advocate for. The second will rely predominantly on a *capacitarian* division of labor where moral responsibility is distributed mostly among actors with different degrees of power – this will be mainly the account that many theorists of global (health) justice prefer. The third scenario is a hybrid division of moral labor that combines both the capacitarian and the functional division of moral labour – the account I argue for here.

The best way to combine both approaches of the division of moral labor is for the capacitarian division of moral labor to primarily apply to global health issues and the functional division of moral labor to primarily apply to domestic health issues. In other words, when we face pandemics and other health emergencies that cannot be solved by one country alone, those with more capacities should also have the greater responsibility to address those challenges. This does not mean that those with less capacity have no moral obligations at all. It only means that the moral responsibility to address global health issues will be proportional to the current abilities of each moral agent, given the immediacy and urgency that such global health emergencies usually require. When we do not face pandemics or any other sort of global health challenges, the moral responsibility to address health issues should fall primarily on individual states whose role is to ensure that such health issues are being taken care of within the boundaries of their jurisdiction and do become global health crises. This does not exclude any type of intervention but limits it in a way that will not be harmful in the long run for local governments.

If we focus only or mostly on the capacitarian division of labor that many theorists of global justice favor, the most important focus will be on international aid that includes health and its determinants sometimes with good reason. But international or development aid that many rich countries have used as their best instrument to discharge their responsibilities for global health can do more harm than good if it does not pay attention to the hybrid approach we suggest. Based on the experience of the last 50 years of development or international aid, the Zambian economist Dambisa Moyo [31]argues in her book *Dead Aid* that international aid has weakened instead of strengthening many African countries both economically and politically. It has done so by depriving these countries of the sense of positive sovereignty that translates into responsibility toward their own people, by inadvertently strengthening corruption networks, by preventing the establishment of a diversified economy, and I will add by increasing the sense of governmental irresponsibility.

In the same vein, the relevance and effectiveness of international or development aid as the best way for rich countries to meet their global (health) justice obligations or for citizens from rich countries to be good in a world of needs has been recently and further questioned by the American philosopher Larry Temkin [32]. A former advocate of effective altruism alongside philosophers like Peter Singer, Temkin has recently expressed serious worries about the possible unintended consequences of the way aid agencies are discharging international aid in the most desperate parts of the world [33]. He shows that international aid, when it is discharged by mainstream aid agencies, can have disastrous consequences by undermining the very capacity of poor countries’ governments to be responsive to their own citizens. This may be the case when aid agencies hire highly qualified staff in poor countries to advance their agenda. On the one hand, this can cause unfair competition for administrative positions, which are generally underpaid, and make the administrative architecture of poor countries less efficient during the period when aid agencies are operating. On the other hand, at the end of their field missions, this can also cause a brain drain that would further weaken the societies that were supposed to be helped. This can also be the case when the interests and priorities of these aid agencies unwittingly take precedence over local priorities and agendas, thus undermining the local autonomy and authority that they are supposed to be strengthening. Finally, this can be the case when external interventions undermine the responsiveness of local authorities and inadvertently lead to government irresponsibility.

Given the record of the international/development aid effectiveness based upon several decades of its implementation, for some (African) scholars, the issue with international aid is no more the conditions of its implementation but may be its existence. What is questioned is not the existence and relevance of rich and powerful Western countries’ obligations to eliminate global poverty or significantly reduce health inequalities. It is the instruments used until now to address such obligations. International aid has been counterproductive in any attempt to meet its goals. One reason for this situation is the excessive reliance on a capacitarian division of labor, where global health responsibilities are mostly assessed through the lens of who can do most. When global health obligations are assessed mostly through the capacitarian division of moral labor, this can put an unfair and unjustifiable burden on rich countries and leave most African countries off the hook regarding their own responsibility for global health.

Likewise, the focus only or mostly on the functional division of moral labor may also be potentially flawed because it will put an unfair burden on African countries and leave some rich countries off the hook regarding their own responsibility for global health. Some interpretation of the functional division of moral labor may suggest that since the primary obligation of any government is toward its own people, health obligations should not be an exception. Even if they are burdened or face some unfavorable circumstances, African societies should still assume their role as the primary agents of justice within the boundaries of their states and be those with the most important obligations to ensure that the basic health needs of their own people are met. Not doing so could be a disservice not only to their sovereignty but also to their sense of responsibility, both of which are needed to better protect the fundamental right to health of their own citizens [34]. Achieving global justice, according to such interpretation, should start at home by strengthening domestic justice, which is the primary responsibility of any government [35]. We are in a better place if everyone properly assumed his or her domestic responsibility [36] and cleaned their house first.

One may take issue and rightly so with the notion of calling ‘everyone to properly assume his or her domestic responsibility’ as this would justify cutting development aid from the West to zero – since none of the Western states can fully assume their own domestic responsibilities. Another worry with such interpretation is that it echoes a key portion of the political agenda of many far-right political parties in the Western world, where the concern is less to see many African countries become autonomous and capable of meeting by their own means their domestic and global health obligations than to ensure that their supposed irresponsibility does not end up changing the face and character of Western nations. The key problem with such an approach is its overreliance on the functional division of labor. Although it is the primary role of any government to be responsive to the basic (health) needs of their own citizens, the imbrication of the local and the global changes the calculus of how far one should stick to such a division of moral labor. It will be unfair for the citizens of African countries, who can claim as all people do a human right to health, to see that right being only an aspiration because their own state cannot realize it.

Given the shortcomings of both the capacitarian or the functional division of moral labor when considered separately, a less-flawed approach that will paint a more accurate picture of the responsibilities of both African and non-African states would be a combination of both approaches of the division of moral labor. Rich countries on the one hand will have more obligations for global health when global health issues or emergencies are at stake. African countries will play a secondary role in making the best possible use of any international assistance that may come along [37]. African countries on the other hand will have more obligations for local health and daily basic health needs that their citizens expect. Rich countries can play a marginal role in helping to reinforce local public health systems.

If we want to achieve global health equity, African societies should therefore stop dumping their duties on rich countries. This happens when the capacitarian division of labor is the predominant moral ground we rely on, and when the clear separation of domestic from global health issues is framed as an impossibility instead of a difficulty that can be overcome, and a constant moving target that can be dealt with. We should therefore morally expect from most African states to address issues that do not require any significant international cooperation to be dealt with properly, with some marginal assistance from rich countries.

Let me conclude this section by saying that we should not give a pass to Sub-Saharan African countries/governments regarding their primary obligations toward their own people. Moreover, we should not condone the idea that, because they are poor, they can dump their duties on wealthy countries simply because those countries are rich. The fact that rich countries are accepting or allowing poor African countries to dump their primary duties towards their own people is not only disrespectful and paternalistic, but also counterproductive. It undermines the political agency of those societies and prevents mature and responsible governments from taking their own moral responsibility seriously, engaging aggressively in capacity-building and discharging their health duties properly. Ensuring that African societies take full responsibility when it comes to the basic health needs of their own people should not prevent us from being vigilant regarding other health obligations that could be wrongly and unjustifiably assigned to them. If African societies have stronger health duties both at the domestic and at the global level, on which ground should we assess them. I turn to this in the next section.

### The ‘potential ability’ argument

The exclusive focus on the current abilities of African societies explains to some extent why the scope of their health obligations towards their own people has been mostly overlooked. But if we look at the potential abilities and the building-capacity process it requires, there is room to be more demanding regarding what African societies ought to do to fully met their health obligations.

In this respect, consider the French Poet Jean de la Fontaine’s Fable ‘The Cicada and the Ant’ [38]. As always with the fables of the brilliant poet Jean de la Fontaine, one can draw various interpretations. This fable has often been presented as celebrating the virtues of hard work or condemning the vice of laziness. It can also be viewed as contrasting human character traits like selfishness versus generosity. But it also raises more philosophical questions: for example, should we help a person who cares little for his own fate? The angle of interests to me in this fable is the way in which it could lead us to rethink the very notion of moral responsibility based on the distinction between current abilities and potential abilities [39] and how this may affect the justification and the scope of the health obligations of African societies. One can assume that before winter, the cicada and the ant had the same ‘potential’ ability, but that at the time of winter, there is a huge difference between the current ability of the ant and the actual ability of the cicada. If such distinction is morally relevant, how should it affect our framing of the health care obligations that African societies have towards their own people?

I suggest that: a) we need to clarify the interesting distinction that exists between the ‘current ability’ and ‘potential ability’ of African societies as moral agents; b) relying exclusively on the current ability of African societies to determine their moral responsibility can be misleading as it provides an incomplete picture of their overall abilities and therefore also of their moral responsibility; c) a full picture will require to address also their potential ability and based on this to morally justify stronger health obligations of African societies; d) moreover, even if one relies solely on the current ability argument, there are still ground to expect stronger health obligations from African societies.

The Potential Ability Argument (PAA) is a version of the ‘ought implies can’ principle. The idea that ‘ought implies can’ suggests, among other interpretations, that moral agents have duties or obligations only where they are able to perform those duties [40]. At first sight, it draws on some theoretical paradoxes. What is the point of recognizing something like health to be a right or even a human right, if there are no suitable moral agents compelled to fulfill it? Knowing that access to healthcare requires significant amounts of resources and given that most sub-Saharan African countries lack those resources *here-and-now*, should we not limit their health obligations to what they can do now which is almost ‘nothing’? It will indeed be counter-intuitive or even cruel to hold African countries morally responsible to provide for the basic health needs of their own people if they simply cannot afford to do so. This will be like asking someone who lacks the ability to swim to jump into the river to save the drowning child or to ask the cicada to feed itself during winter when she simply cannot. We do believe that where people are unable to fulfil a specific duty, they have no moral responsibility to fulfil it. The child will still be in a position where someone else has the duty to save her from drowning but performing such an obligation may either fall on somebody else or require the pedestrian to do something else than swimming herself. It should not matter at all who should end up saving the child (his own parents or a pedestrian stranger). What matters most is to save the child. Likewise, it should not matter primarily who should provide for each African citizen to have her basic health need met or if the moral responsibility to provide for basic health needs of African citizens falls on African governments first. What matters most is to ensure each African citizen has her basic health needs covered, primarily by rich countries if they are in best position to do so. In conclusion, wherever people have the capacity to fulfil an obligation, they have a strong obligation to do so. Wherever people do not have a capacity to fulfil an obligation, they simply do not have the obligation to fulfil it.

Most often, the ‘ought implies can’ principle refers to the *here-and-now-ability* of a specific agent: its ‘current ability’. Looking at the current ability of African societies, the lack of financial resources is notable. Despite some progress, most African countries find themselves at the bottom of most rankings and survey regarding poverty, health care systems or educational systems [41]. This is important because the lack of financial means seriously affect the ability of setting up any meaningful public health policy. As Sunstein and Carr show very well, the protection of freedom and of rights, including the right to health, heavily depends on taxes or financial revenues [42]. This is true for many African societies where the lack of financial resources means lack of enough hospital beds, few medical personnel to be hired, fewer mosquito nets than needed, etc. Moreover, a poor educational system also means poor literacy regarding prevention of diseases like sexually transmissible infections or cleaning of hands and environments where people live. Based on this picture of the current ability of African societies, the temptation would be to conclude that African societies are like the pedestrian who cannot swim or the cicada who cannot feed itself when winter comes.

But, even if it is true that many African countries are dealing with many competing needs that affect their current ability to properly discharge their health duties today, that is an incomplete picture of their overall ability. Recall La Fontaine’s fable. The cicada had the potential ability to collect grains of wheat and barley and store them to prepare for winter but voluntarily failed to do so. Even if what matters most is to ensure that African citizens today have their basic health needs met, one cannot be insensitive to the way some countries build or refuse to build the necessary ability to discharge their obligations when assessing the obligations of moral agents. This opens the door to scrutinizing if many African societies had the potential ability in the past to discharge their obligation today and failed to do so.

One could say, as some economists have suggested, that some African countries were at the same economic level than some Asian countries that are far richer than they are today [43], and therefore would have been best positioned to meet their current domestic health obligations. These economists have rightly pointed out the difference between the economic potential of what a country like the Democratic Republic of Congo possesses with its huge natural resources compared for example to Japan, and the actual economic disaster it is in today. Had they taken another path economically such countries may not be in a position where they lack the financial power to meet their health obligations, and the conversation about their health obligations and about global health inequalities more broadly would be completely different?

The economic missteps of many African societies also echo their political missteps, especially if one agrees with Amartya Sen and many economists that what makes a country rich is less its economic fabric than its political infrastructure [44]. In this respect, many African countries abandoned the democratic feature of their political systems pre-independence [45] and adopted unfair and authoritarian regimes that prevented them from becoming richer and building the economic (production) and political (distribution) ability to meet their health obligations today. One can rightly push back against the Africa-Asians dragon’s comparison and say that the economic circumstances that made it possible for these Asian countries to prosper were not fully available to many African countries. Although microeconomic, macroeconomic, and political factors did play a role in where both sets of countries diverged at some point [46], we cannot discount the long-term negative and corrosive impact that the colonial legacy, in addition to centuries of slavery, played into preventing the capacity building process of many African countries [47].

Nevertheless, scrutinizing what African societies could have done economically and politically to be better off today does not matter only to recount the past. It does matter because the discussion about their potential ability is an ongoing one, i.e., they still have the potential ability today to discharge their health obligations tomorrow and are currently failing to do so. Consider in this respect, the notions of ‘progressive realization’ of so-called socio-economic rights like the right to health which is entrenched in the human rights discourse and the notions of ‘developing capacity’ or ‘building capacity’ often associated with international development policies. For example, the United Nations’ International Covenant on Economic, Social and Cultural Rights requires (African) States to “take steps, individually and through international assistance and co-operation, especially economic and technical, to the maximum of [their] available resources, with a view to achieving progressively the full realization of the rights recognized in the present covenant by all appropriate means, including particularly the adoption of legislative measures” [48]. Whereas the United Nations Division for Sustainable Development Goals suggests that capacity building “encompasses the country’s human, scientific, technological, organizational, institutional and resource capabilities. A fundamental goal of capacity-building is to enhance the ability to evaluate and address the crucial questions related to policy choices and modes of implementation among development options, based on an understanding of environmental potentials and limits and of needs as perceived by the people of the country concerned” [49].

Both notions encompass two key elements of the PAA: resources and time. First, as already said, the lack of current resources makes it impossible to require African societies to fully meet their health obligations now. If the pedestrian who is unable to swim now cannot be forced to jump into the river to save the drowning child, she can at least be forced to learn how to swim given the high probability that there will be many potential drowning children in the future. Learning how to swim or building capacity will take some time, but it is a moral requirement, not an optional path and that is why the covenant call for states to submit target goals to building their capacity to fully realize fundamental socioeconomic rights including the human right to health.

In view of this, both notions convey the idea that states, even poor, have the immediate responsibility to provide for the health of their own people within the constraints of their current available resources and they must build and expand their ability to meet their obligations going forward. Most African societies should therefore be accountable for what they are failing to do now to ensure their own people have their basic health needs met. ‘Progressive realization’ is not therefore a scapegoat to avoid taking immediate steps toward building or developing their future ability to meet their health obligations.

If the notions of ‘progressive realization’ and ‘building capacity’ suggest stronger moral obligations from African states, they also emphasize two challenges that should be addressed regarding the proper role of international assistance. First, what should be the fate of the realization of the human right to health of African citizens when their societies are still building their capacities, especially if international aid can do more harm than good? Second, how can African societies really build any relevant current abilities if they bend under the yoke of an international economic order which, through debt servicing, for example, prevents them from effectively developing the current abilities they need to be fully and effectively responsive of the basic health needs of their people?

Regarding the first challenge, there is a need to rethink the way international assistance or solidarity in the domain of health should operate. We know that international assistance that focus primarily on material and financial transfers can do more harm than good. It does more harm than good because it weakens the very capacity African societies need to develop to meet their health obligations in the long run. The fact that international assistance that focuses primarily on material and financial transfer should stop does not mean that rich countries stop having strong moral obligations to assist poor countries. It means only that the primary focus of international assistance or solidarity should no more be the transfer of material or financial transfers, but how to best contribute to the capacity-building process of many African societies. Giving such priority to capacity-building is also the best bet that many aspects of the human right to health that many African citizens can claim will be well protected during that phase of progressive realization.

If the priority of international assistance or solidarity is on capacity-building instead of material and financial transfers which are quickly engulfed in networks of corruption or caught in the vice of waste and poor governance, this can also help addressing the second challenge. Very often, the nature of international assistance from rich countries to poor countries is a zero-sum operation. What is given by the right hand - transfers of material and money - is very often taken back by the left hand - exorbitant interest on the financial debt of poor countries. The servicing of debt is a major obstacle to the capacity-building process of many African countries. It is a sword of Damocles which not only contributes to keeping African countries dependent on rich countries but above all deprives them of room to maneuver that can enable them to develop the capacities necessary to fulfill their health obligations. I have argued elsewhere how unfair the financial debt – which is an odious debt – is, that African countries owe to rich countries, especially given how vulture funds operate following the restructuring of the sovereign debt of many African countries [50]. Any relevant international assistance or solidarity that focuses on contributing primarily to the capacity-building process of African countries should ensure that the servicing of debt does not impede the journey African countries should take to transform their potential ability to current abilities. This is the part of the moral labor that rests on rich countries and which is not under the control of any African countries. Despite much-reduced room to maneuver, there is still room to hold many African countries more accountable for their part of the moral labor that remains under their control.

If the distinction between potential and current abilities provides some moral ground to expand the scope of the health obligations of African societies towards their own people, it can also face some of the challenges just mentioned. Nevertheless, even if we focus solely on the current abilities of African societies, there are some actions/inactions and decisions of their own making that prevent many African states them from meeting their health obligations towards their own people. One can imagine, for example, that some countries could significantly reduce the State’s car fleet and other useless expenses to devote them to the construction of hospitals or the increased training of health personnel. These countries could allocate less financing to the defense budget, refrain from financing useless wars, and devote this money instead to financing the local clinical research necessary to address the pathologies most present locally. These are decisions that are within the reach of African political leaders themselves today and for which they should be held accountable. Even regarding situations that do not directly depend on them like the servicing of debt, there is still the possibility for these African States to regroup, for example, and to use the few geopolitical margins of maneuver available to them to put pressure on those rich States whose decisions have the consequence of compromising their current and potential abilities to meet their health obligations towards their own people.

## Conclusion

I have provided a theoretical agenda challenging the implicit minimization by most Western global justice theorists of the health obligations of Sub-Saharan African states. By arguing that richer countries that can assist poorer countries in need should do so, those scholars implicitly assume that poor countries should have weaker obligations towards their citizens. Against this claim, I have attempted to show that it should be the primary responsibility of each state and therefore of each Sub-Saharan African state to ensure that the fundamental rights of its own citizens are protected, including health, and that most states – including Sub-Saharan Africa’s –have the ability and resources to protect fundamental rights of the type violated in the Monique case. Hence, Sub-Saharan African states should build their ability to address other health challenges that go beyond Monique-type cases. In this respect, the assessment of their health obligations shouldn’t rely solely on what they are able to do today (current ability) but should include also all the steps they are failing to take right now to build their capacity to be fully responsive to the health needs of their citizens in the future (potential ability).

The health situation in Sub-Saharan Africa and the failure to frame the health obligations of African countries properly only mirror what is more generally problematic with our practice of political philosophy applied to global issues today. The temptation by most Western scholars to discount at worst, or downplay at best, the specific (health) obligations of African countries disserve the cause of effectively advancing global justice, and those scholars should rethink how they better include other non-Western perspectives in their own thought process. As this paper was purposefully also an attempt to present an ‘African perspective’ on these issues, it cannot emphasize enough the necessity for non-Western and especially African political theorists to take the lead and develop their own account of what global justice should look like when it comes to health issues and how it should interact with domestic justice in Africa. Addressing the issues described in this paper requires academic responsibility beyond government responsibility in Sub-Saharan Africa.

Finally, although my primary focus was to shed some light on the specific health responsibility of Sub-Saharan African states toward their own people, one cannot elude the influence of other ‘external’ factors for a full and equitable account of African states’ responsibility. If African states should have the primary responsibility of providing for the health of their own people, they can however see their effort to build or develop their capacities undermined by external factors like the odious debt that puts an unfair burden on their economies, which are the consequences of us living in a globalized economy. Further research is therefore needed to elaborate on the proper role wealthier nations can play once it is acknowledged that African states should bear the primary responsibility to protect and fulfill the fundamental rights, including the human right to health, of their own people. An interesting exploration based on the arguments proposed here will be to fully understand what Sub-Saharan African states really need in their struggle toward building their capacity to be responsive to their people’s health needs. Rich countries should stop any (financial) international aid that run the risk to replace and play the noxious role of natural resources. Instead, rich countries should refrain from abusing their dominant position in designing an unfair global economic order. Such unfair economic order prevents fragile African economies from truly emerging, adding unnecessary and additional hurdles in their struggle to build the capacities they need – efficient economies and reliable political institutions – to meet their obligations towards their own people.

## Data Availability

Not applicable.
